# Antimicrobial Photodynamic Therapy Mediated by Fotenticine and Methylene Blue on Planktonic Growth, Biofilms, and Burn Infections of *Acinetobacter baumannii*

**DOI:** 10.3390/antibiotics11050619

**Published:** 2022-05-04

**Authors:** Lívia M. A. Figueiredo-Godoi, Maíra T. Garcia, Juliana G. Pinto, Juliana Ferreira-Strixino, Eliseu Gabriel Faustino, Lara Luise Castro Pedroso, Juliana C. Junqueira

**Affiliations:** 1 Institute of Science and Technology (ICT), São Paulo State University (Unesp), São José dos Campos, São Paulo 12245-000, Brazil; maa.terra@hotmail.com (M.T.G.); eliseugf@icloud.com (E.G.F.); lara.luise@unesp.br (L.L.C.P.); juliana.junqueira@unesp.br (J.C.J.); 2 Photobiology Applied to Health (Photobios), University of Vale of Paraiba/UNIVAP, São José dos Campos, São Paulo 12244-000, Brazil; jgbiomd@gmail.com (J.G.P.); juferreira@univap.br (J.F.-S.)

**Keywords:** *Acinetobacter baumannii*, photodynamic therapy, chlorin, fotoenticine, methylene blue, burns, *Galleria mellonella*

## Abstract

Antimicrobial photodynamic therapy (aPDT) is considered a promising alternative strategy to control *Acinetobacter baumannii* infections. In this study, we evaluated the action of aPDT mediated by a new photosensitizer derivative from chlorin e-6 (Fotoenticine—FTC) on *A. baumannii,* comparing its effects with methylene blue (MB). For this, aPDT was applied on *A. baumannii* in planktonic growth, biofilms, and burn infections in *Galleria mellonella*. The absorption of FTC and MB by bacterial cells was also evaluated using microscopic and spectrophotometric analysis. The results of planktonic cultures showed that aPDT reduced the number of viable cells compared to the non-treated group for the reference and multidrug-resistant *A. baumannii* strains. These reductions varied from 1.4 to 2 log_10_ CFU for FTC and from 2 log_10_ CFU to total inhibition for MB. In biofilms, aPDT with MB reduced 3.9 log_10_ CFU of *A. baumannii*, whereas FTC had no effect on the cell counts. In *G. mellonella*, only MB-mediated aPDT had antimicrobial activity on burn injuries, increasing the larvae survival by 35%. Both photosensitizers were internalized by bacterial cells, but MB showed a higher absorption compared to FTC. In conclusion, MB had greater efficacy than FTC as a photosensitizer in aPDT against *A. baumannii*.

## 1. Introduction

*Acinetobacter baumannii* is a Gram-negative coccobacillus belonging to the Moraxellaceae family, being among the main opportunistic pathogens associated with nosocomial infections [1,2,3]. The growth of *A. baumannii* in hospital environments can be attributed to its ability to survive on abiotic surfaces for long time periods and to acquire resistance to many antibiotic classes [1,3,4]. *A. baumannii* can also colonize the human skin for several months [4], becoming a microbial source for local and systemic infections, mainly in severely ill patients [5]. Due to the high resistance profile, the treatment of *A. baumannii* infections in debilitated patients has been performed with carbapenems (imipenem and meropenem), which are beta-lactam antibiotics with a wide spectrum of action, considered one of the “last resort” drugs for these patients [6,7,8]. However, in the last decades, there has been a significant increase in the prevalence of carbapenem-resistant *A. baumannii* (CRAB), placing this microorganism on the antibiotic-resistant priority pathogens list of the World Health Organization (WHO) that urgently needs new therapies [9,10].

In this context, antimicrobial photodynamic therapy (aPDT) has been considered a promising strategy for controlling multi-drug-resistant bacterial infections [11,12,13,14,15,16,17,18]. aPDT consists of the administration of a non-toxic photosensitizer, followed by irradiation by visible light with a wavelength capable of activating the photosensitizer in the presence of molecular oxygen. This reaction results in the formation of reactive oxygen species (ROS) that kill the bacterial cell via a multi-target mode of action instead of the key-lock principle of conventional antibiotics. Since aPDT acts on several molecular targets (proteins, lipids, and nucleic acids), the development of resistance by bacterial cells is very improbable [19]. Seeking to increase the efficacy of aPDT, several photosensitizers have been investigated in the last years, such as phenothiazines, hematoporphyrin derivatives, chlorins, xanthenes, phytolocyanins, and curcumin [20]. 

Certainly, the phenothiazine methylene blue has been the most used photosensitizer until now. This photosensitizer has received regulatory approval in several countries around the world for use in aPDT. However, phenothiazine dyes are not highly active, and many laboratories have tried to introduce new compounds with better photodynamic properties [21]. Among the new photosensitizers, fotoenticine (FTC), a derivative from chlorin e-6, has shown high photodynamic activity against Gram-positive bacteria [22]. Chlorines are second-generation photosensitizers that belong to the class of tetrapirrolic substances and can be activated by light at 650-800 nm, providing possibilities to treat a variety of infections, including deeper lesions [23]. Although chlorine is a very promising photosensitizer, there are no studies that investigated the use of fotoenticine in the control of *A. baumannii*. 

Besides the identification of new photosensitizers, there is a necessity to thoroughly investigate the action mechanisms of aPDT on in vivo models as its effectiveness also depends on the light penetration in the tissues and the response of the immune system [24]. Seeking to increase the number of in vivo studies and at the same time reduce the use of vertebrate animals, the *Galleria mellonella* invertebrate model has gained increasing recognition by the scientific community. The success of *G. mellonella* as a host model can be attributed to the achievement of results comparable to vertebrate animals, ease and low cost of rearing, short life cycle, ethical acceptance, and presence of humoral and cellular immune responses [25,26]. These advantages, associated with the recent sequencing of its genome, make *G. mellonella* a reliable and very useful in vivo model [27,28]. Previously, our group introduced the use of *G. mellonella* as a study model for aPDT by injecting both pathogen and photosensitizer into the hemolymph of these animals [24,29,30]. In the present study, we proposed a burn infection model that can be used in aPDT studies focused on bacterial skin infections.

Thus, the objective of this study was to evaluate the effects of aPDT mediated by FTC or methylene blue (MB) on *A. baumannii* strains, comparing its effects on planktonic growth, biofilms, and experimental local infections in the *Galleria mellonella* model. In addition, the capacity of both photosensitizers to be absorbed by *A. baumannii* cells was assessed. 

## 2. Results

### 2.1. Effects of aPDT on Planktonic Cultures of A. baumannii Strains

For the study of planktonic cultures, three strains of *A. baumannii* were analyzed, including a reference strain (ATCC 19606) and two multidrug-resistant clinical strains (A1 and A4). Planktonic cultures of all the strains were submitted to six different experimental conditions, forming the following groups: fotoenticine and light (FTC+L+ group), methylene blue and light (MB+L+ group), fotoenticine without irradiation (FTC+L- group), methylene blue without irradiation (MB+L- group), light in the absence of photosensitizer (P-L+ group), and absence of photosensitizer and light (P-L- group). After each treatment condition, the number of viable cells of *A. baumannii* was determined by counting the number of colony-forming units (CFU).

**Figure 1:**
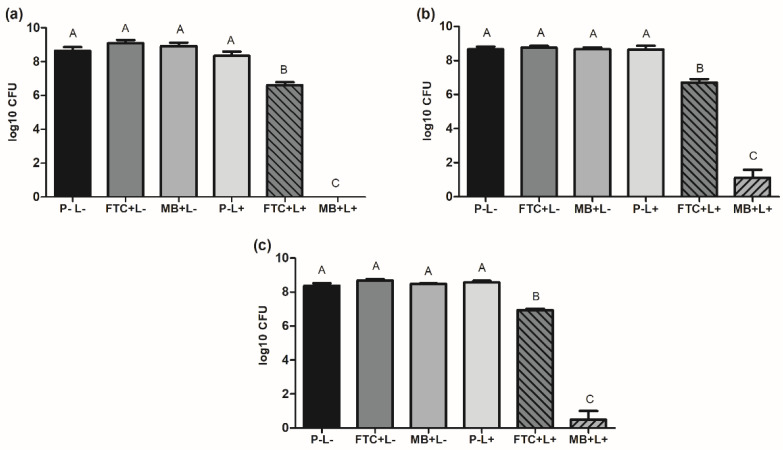
Means and standard deviation of viable cells of *A. baumannii* (log_10_ CFU) in planktonic growth for the following groups: absence of photosensitizer and light (P-L-), FTC without light (FTC+L-), MB without light (MB+L-), absence of photosensitizer with light (P-L+), FTC with light (FTC+L+), and MB with light (MB+L+). (**a**) *A. baumannii* reference strain (ATCC 19606). (**b**) *A. baumannii* A2 clinical strain. (**c**) *A. baumannii* A4 clinical strain. ANOVA and Tukey test: different letters represent statistical difference between the groups (*p* ≤ 0.05).

For all the studied strains, the groups P-L-, FTC+L-, MB+L-, and P-L+ showed similar numbers of viable cells that ranged from 8.3 to 9.0 log_10_ CFU, with no statistical differences between the groups. These results indicated that the use of FTC or MB in the dark had no toxic effects on *A. baumannii* cells. On the other hand, the groups treated with aPDT mediated by fotoenticine (FTC+L+) or methylene blue (MB+L+) presented a significant reduction in the number of viable cells compared to the control group (P-L-). For the reference strain, a reduction of 2.0 log_10_ CFU in the FTC+L+ group and a total inhibition of *A. baumannii* cells in the MB+L+ group was observed. For the clinical strains, aPDT with FTC (FTC+L+) led to bacterial reductions of 1.4 to 1.9 log_10_ CFU, while the aPDT with MB (MB+L+) reached reductions of 7.5 to 7.8 log_10_ CFU (Figure 1). These data showed that all the strains of *A. baumannii* in planktonic growth were susceptible to aPDT mediated by FTC; however, these effects were less pronounced when compared to aPDT mediated by MB. 

### 2.2. Effects of aPDT on A. baumannii Biofilms

**Figure 2:**
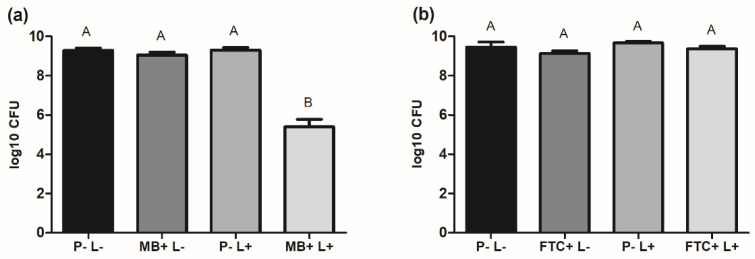
Means and standard deviation of viable cells of *A. baumannii* (log_10_ CFU) in biofilms for the following groups: absence of photosensitizer and light (P-L-), FTC without light (FTC+L-), MB without light (MB+L-), absence of photosensitizer with light (P-L+), FTC with light (FTC+L+), and MB with light (MB+L+). (**a**) Experiment performed to test the photosensitizer MB. (**b**) Experiment performed to test the photosensitizer FTC. ANOVA and Tukey test: different letters represent statistical difference between the groups (*p* ≤ 0.05).

Biofilms of *A. baumannii* were formed on microtiter plates for 48 h using the reference strain (ATCC 19606). After that, the mature biofilms were divided into 6 groups and submitted to different treatment conditions as described above (P-L-, FTC+L-, MB+L, P-L+, FTC+L+, and MB+L+ groups). The results of viable cells count showed a biofilm formation of approximately 9.3 log_10_ CFU for the groups not submitted to aPDT, including the P-L-, FTC+L-, MB+L-, and P-L+ groups. Interestingly, the treatment with MB-mediated aPDT caused a significant inhibition in the biofilms, with 3.9 log_10_ CFU of reduction when compared to the control group (P-L-). However, no inhibition in the number of viable cells was found in the group treated with FTC-mediated therapy (Figure 2), indicating that the *A. baumannii* biofilms were resistant to the antimicrobial photodynamic effects of FTC. 

### 2.3. Effects of aPDT on Burn Infections in Galleria Mellonella Model 

After in vitro studies in planktonic cultures and biofilms of *A. baumannii*, we moved to an in vivo model of burn infection in G. mellonellla. For this study, *G. mellonella* larvae were injured with a heated steel instrument in their cuticle to induce a burn wound. Immediately after, this lesion was locally infected with a suspension of *A. baumannii* ATCC 19606). After 30 min, the photosensitizer was administrated to the lesion. Then, 30 min later, the burn wound was treated with aPDT mediated by FTC or MB. The effects of aPDT on *G. mellonella* were evaluated for 5 days to determine the survival curve and health index. 

#### 2.3.1. Survival Curve

**Figure 3:**
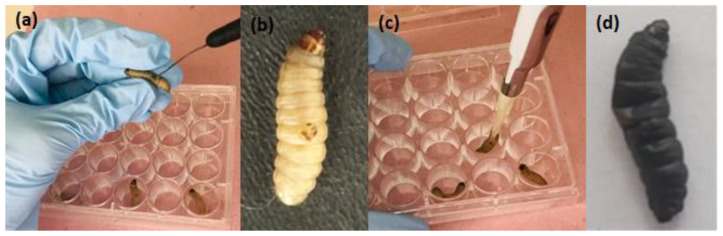
Burn infection model in *G. mellonella*. Larvae were injured with a heated steel instrument in their cuticle (**a**), resulting in a burn lesion (**b**). After larvae were locally infected with a suspension of *A. baumannii* (**c**), it was possible to observe the progression of systemic infection (**d**).

**Figure 4:**
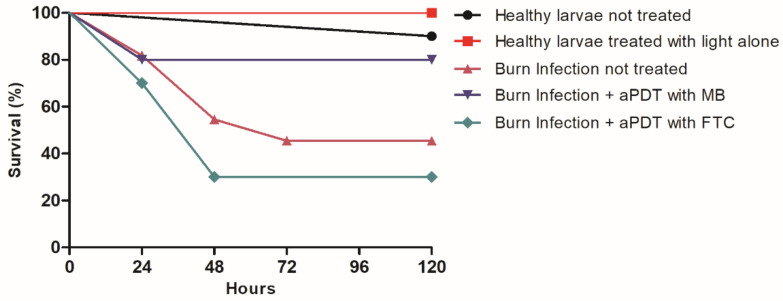
Survival curve of *G. mellonella* obtained in the following groups: healthy larvae not treated, healthy larvae treated with light alone, larvae with burn infected by *A. baumannii* and not treated, larvae with burn infected by *A. baumannii* and treated with MB-mediated aPDT, and larvae with burn infected by *A. baumannii* and treated with FTC-mediated aPDT. Comparison of survival curves by Log-rank test: no statistically significant differences were found for the groups “burn infection + aPDT with MB” (*p* = 0.1827) and “burn infection + aPDT with FTC” (*p* = 0.5780) in relation to “burn infection not treated”.

At the end of the experiment (5 days after infection), the larvae not treated with aPDT presented a survival rate of 45%, suggesting that the burn wound model infected by *A. baumannii* was able to cause a systemic infection that killed the larvae (Figure 3). Promisingly, in the larvae treated with MB-mediated aPDT, the survival rate increased to 80%, showing that this therapy was capable of protecting *G. mellonella* larvae from subsequent systemic infection. On the other hand, the treatment with FTC-mediated aPDT resulted in only 30% of survival (Figure 4). Thus, considering the parameters employed in this study, only aPDT mediated by MB proved to be effective in treating the infection by *A. baumannii* in burn wounds in *G. mellonella*.

#### 2.3.2. Health Index

**Figure 5:**
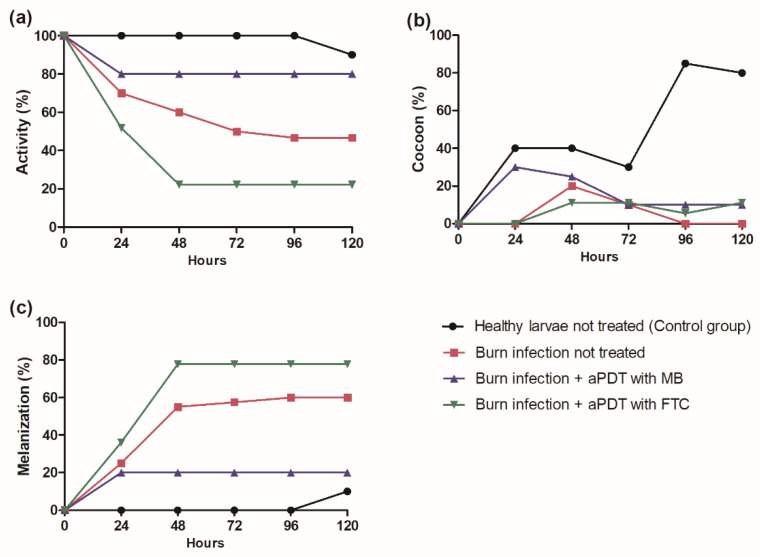
Means of scores obtained in the health index analysis for the groups: healthy larvae not treated (control group), burn infection not treated group, burn infection + aPDT with MB group, and burn infection + aPDT with FTC group. ANOVA and Tukey test (*p ≤* 0.05). (**a**) Movement activity: burn infection not treated group compared to burn infection + aPDT with MB group (*p* = 0.2668) and burn infection + aPDT with FTC group (*p* = 0.2321). (**b**) Cocoon formation: burn infection not treated group compared to burn infection + aPDT with MB group (*p* = 0.8055) and burn infection + aPDT with FTC group (*p* = 0.9989). (**c**) Melanization: burn infection not treated group compared to burn infection + aPDT with MB group (*p* = 0.1713) and burn infection + aPDT with FTC group (*p* = 0.6183).

In addition to the calculation of the survival curve of *G. mellonella*, the health status of the larvae was also monitored, considering the attributes of movement activity, cocoon formation, and melanization. Regarding the movement activity (Figure 5a), at the end of the experiment, the control group formed by healthy larvae not treated showed 90% of activity, while the burn infection group had 46% of activity. Interestingly, the larvae subjected to burn infection and treated with MB-mediated aPDT maintained 80% of movement activity, but the treatment with FTC-mediated aPDT showed only 22% of activity. 

The second attribute analyzed was cocoon formation (Figure 5b). The healthy larvae control group had an index of 80%, with a statistically significant difference (*p* = 0.0036) in relation to the burn infection not treated group (index of 0%). Larvae subjected to burn infection and treated with aPDT mediated by MB or FTC obtained 10% of cocoon formation, with no significant difference in relation to the burn infection not treated group. 

Lastly, in the analysis of melanization (Figure 5c), an index of 10% was found for the healthy larvae control group and 60% for the burn infection not treated group, with a statistically significant difference between them (*p* = 0.0145). When the larvae with burn infections were treated with aPDT mediated by FTC, the melanization index remained elevated (77%). However, the treatment with aPDT mediated by MB led to a significant reduction of melanization, with an index of 20%.

In summary, the inoculation of *A. baumannii* in the burn injury reduced the movement activity and cocoon formation of larvae, as well as increased the melanization rate, characterizing the normal infection process. With the application of MB-mediated aPDT, it was possible to increase the movement and cocoon formation of larvae and promote the reduction of the melanization process. Considering all the attributes, the general health index was 9.4 for the healthy larvae group, 4.4 for the burn infection not treated group, 7.4 for burn infection + aPDT with MB group, and 2.8 for burn infection + aPDT with FTC group. 

### 2.4. Absorption of Photosensitizers by the A. baumannii Cells

To investigate the action mechanisms that could be evolved in the different results obtained in relation to the susceptibility of *A. baumannii* to aPDT mediated by MB or FTC, we added assays to evaluate the photosensitizers’ absorption by bacterial cells. These studies were performed with A. baumannii reference strain (ATCC 19606) in planktonic growth not subjected to aPDT, using a qualitative analysis of confocal microscopy and quantitative analysis of absorbance by spectrophotometry. 

#### 2.4.1. Analysis of Confocal Microscopy

**Figure 6:**
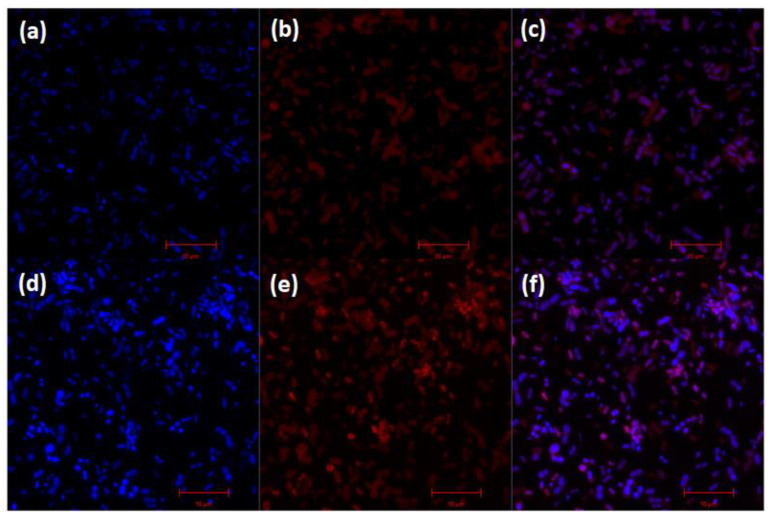
Confocal microscopy of MB and FTC internalization by *A. baumannii* planktonic cells. (**a**,**d**) *A. baumannii* cells with DAPI stained; Internalization of MB (**b**) and FTC (**e**) by *A. baumannii* cells; The two overlapping images for MB (**c**) and FCT (**f**).

In the confocal microscopy images obtained for the *A. baumannii* treated with MB or FTC, it was possible to observe the morphology of the bacterial cells through the DAPI stained in blue (Figure 6a,d), the presence of the photosensitizers inside the cells in red (Figure 6b,e) and the two overlapping images (Figure 6c,f). In view of these results, it was found that both MB and FTC were capable of internalizing into the *A. baumannii* planktonic cells.

#### 2.4.2. Test of Absorbance by Spectrophotometry

**Figure 7:**
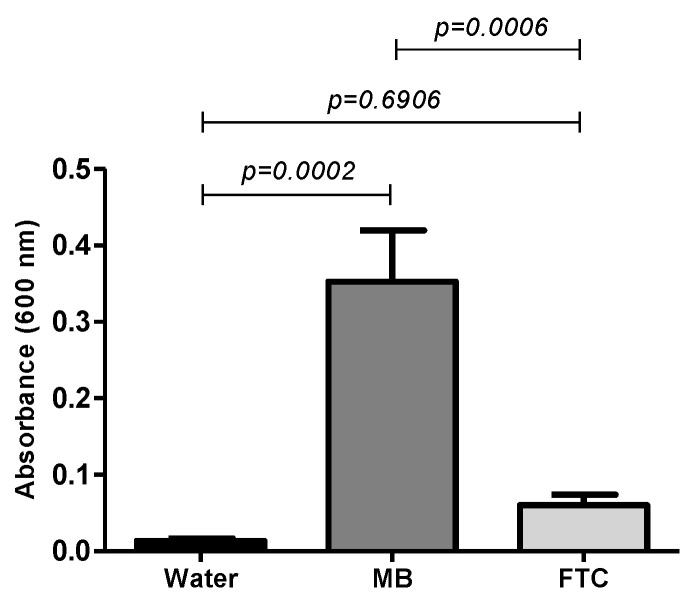
Means and SD of absorbance values (600 nm) in optical density values showing the absorption of MB and FTC by the *A. baumannii* cells (ATCC 19606). (Water) *A. baumannii* in sterile distilled water only; (MB) *A. baumannii* treated with MB; (FTC) *A. baumannii* treated with FTC.

The quantitative analysis of photosensitizers’ absorption by spectrophotometry confirmed that both MB and FTC were able to penetrate *A. baumannii* cells (Figure 7). However, the group treated with MB showed higher absorption values (OD = 0.352) than the group treated with FTC (OD = 0.060), with a statistically significant difference between the groups (*p* = 0.0006). These results suggested that the greater absorption of MB by A. baumannii cells in relation to FTC can be associated with the higher efficacy of MB-mediated aPDT in both in vitro and in vivo studies. 

## 3. Discussion

Antimicrobial photodynamic therapy has shown potential to be used as an adjuvant therapy against bacterial infections; however, its success is dependent on the efficiency of the photosensitizer employed [31]. This study proposed the use of a new chlorine e-6 (FTC) not yet tested as a photosensitizer for aPDT in the control of *A. baumannii,* comparing its effects with the conventional photosensitizer MB. 

First, we verified that aPDT mediated by FTC or MB was able to decrease the *A. baumannii* cells in planktonic cultures when both reference and multidrug-resistant strains were evaluated. aPDT associated with FTC caused reductions varying from 1.4 to 2 log_10_ CFU of *A. baumannii* cells, whereas MB-mediated aPDT reached 2 log_10_ CFU reduction to total inhibition. These results showed a greater antibacterial effect of MB in aPDT on *A. baumannii* cells compared to FTC. Sabino et al. [32] evaluated the effect of aPDT with MB on multidrug-resistant strains of Gram-negative and Gram-positive bacteria in planktonic cultures. Using a visible red-light source with an energy density of 40 J/cm^2^ and irradiation time of 75 s, these authors found more than 5 log_10_ CFU reduction for strains of *A. baumannii*, *Escherichia coli*, *Enterococcus faecium*, *Enterococcus faecalis,* and *Staphylococcus aureus*. For strains of *Klebsiella aerogenes*, *Klebsiella pneumoniae,* and *Pseudomonas aeruginosa*, the same microbial reduction levels occurred with 7 min of irradiation. In another study, Mello et al. [33] applied aPDT mediated by MB on carbapenem-sensitive and carbapenem-resistant *A. baumannii* isolates. Using a red-light source with 39.5 J/cm^2^, aPDT decreased the number of *A. baumannii* planktonic cells for all isolates tested, reaching bacterial reductions ranging from 63 to 88% for susceptible isolates and from 26 to 97% for resistant isolates. These studies confirm that aPDT has antibacterial activity against multidrug-resistant pathogens, including *A. baumannii.* However, the inhibition level can vary according to the aPDT parameters, species, and strains analyzed. Promisingly, regardless of differences in the light sources, types of photosensitizers, and microbial taxonomy, aPDT shows antibacterial effects against multidrug resistant strains. 

Next, aPDT using FTC or MB was tested in biofilms of *A. baumannii* reference strain. For both photosensitizers, the cells of *A. baumannii* in biofilms had reduced susceptibility to aPDT compared to its planktonic growth. For the photosensitizer MB, aPDT led to a total inhibition of planktonic cells and a reduction of 3.9 log_10_ CFU of biofilms. In relation to the photosensitizer FTC, it was found a reduction of 2 log_10_ CFU of planktonic cells; however, there was no inhibition in biofilms. These results corroborate previous studies that show lower susceptibility of microorganisms enclosed in biofilms than in planktonic states [34,35]. As photosensitizers and oxygen are important components for aPDT reaction, the reduced susceptibility of biofilms may be associated with the limited diffusion of photosensitizers and the hypotoxic microenvironment of biofilms [35].

In both planktonic and biofilm studies, aPDT mediated by MB had higher antimicrobial activity compared to FTC. These results were divergent from the study of Garcia et al. [36], in which MB and two photosensitizers derived from chlorin, Photoditazine^®^ (PDZ) and FTC, were tested on the *Streptococcus mutans* biofilm. aPDT caused a microbial reduction of 4 log_10_ CFU with MB, 6 log_10_ CFU with PDZ, and complete elimination with FTC, indicating that aPDT mediated by photosensitizers derived from chlorin had greater antimicrobial action on *S. mutans* biofilm than MB. Probably, the differences in the results of FTC-mediated PDT on *S. mutans* and *A. baumannii* may be attributed to the cell wall composition of Gram-positive and Gram-negative bacteria. The outer membrane of Gram-negative bacteria, which confers more resistance to antimicrobial drugs, also limits the entry of the photosensitizer into the bacterial cell [19]. Some alternatives to improve the action of photosensitizers on Gram-negative bacteria have been proposed, such as nano-drug delivery systems [35,37]. Among the various nanoparticles available, chitosan nanoparticles show advantages as they are biodegradable, non-toxic, and have a low immunogenic index [38]. Chitosan is a natural cationic biopolymer [39], so the photosensitizer encapsulated by chitosan nanoparticles (with positive charges) may more easily adhere to the cellular wall of the Gram-negative bacteria and consequently exert higher antimicrobial efficacy [38]. Possibly, a nano-drug delivery system can be a strategy to improve the action of FTC-mediated aPDT on *A. baumannii*.

After in vitro tests, the response of *A. baumannii* to aPDT mediated by FTC or MB was analyzed using a new in vivo model of burn infection in *G. mellonella*. Until now, most of the studies with burn infections were developed using murine models. These models usually require many animals and a long period of time [40]. In addition, ethical restrictions that seek to reduce the use of vertebrate animals have stimulated the development of new infection models in invertebrate animals. In this study, we established a burn model in *G. mellonella* larvae to reproduce superficial infections by *A. baumannii* that can be locally treated with aPDT. The model was adjusted and established based on the invertebrate burn protocol recently developed by Maslova et al. [41]. After inducing a burn injury on the back of the larvae, the infection was performed with topical administration of *A. baumannii* suspension. After 30 min, the photosensitizer (FTC or MB) was applied and then irradiated.

The effect of aPDT on burn injuries infected by *A. baumannii* was analyzed by the survival curve of *G. mellonella* in 5 days. The group with burn infection not treated showed a mortality rate of 55%. The application of MB-mediated aPDT reduced the mortality rate to 20%. However, FTC-mediated aPDT did not improve the larvae survival. These data showed that only the use of MB in the aPDT provided an improvement in the burn infections in the *G. mellonella* model. Until now, there are no studies of aPDT application in wound infections in *G. mellonella,* and our results can be discussed only with studies using murine models. Dai et al. [42] developed a model of burn injuries in mice infected with *A. baumannii*. The microorganism inoculation was carried out in the burn lesion located on the mouse’s dorsum 5 min after the burn injury. The aPDT consisted of the topical application of the photosensitizer, which was a covalent conjugation of chlorin (e6) to polyethylenimine, after 30 min, 24, and 48 h of infection, followed by illumination with a visible red-light source. aPDT applied immediately after infection resulted in a bacterial reduction of 3 log_10_ CFU, while applications 1 or 2 days after infections caused reductions of about 1.7 log_10_ CFU. The authors attributed the success of aPDT due to the conjugation of chlorin e-6 to the cationic polymer polyethylenimine, which can facilitate the penetration of FS in Gram-negative bacteria. 

To complement the survival assays of *G. mellonella*, analysis of additional health attributes, such as movement activity, melanization, and cocoon formation, have been used as important information to assess in more detail the virulence of microorganism and infection development [43]. The movement and cocoon formation indices are attributes of a healthy larva, while the melanization index is related to the immune response to the infection by a pathogen. In the present study, the burn infection reduced the movement activity and cocoon formation of larvae, as well increased the melanization, characterizing an expected infection process. According to Wand et al. [44], more virulent strains of *A. baumannii* generate higher rates of melanization in *G. mellonella* larvae, usually occurring after 4 h of infection and signaling a larval death within 24 h. The reduction of cocoon formation may correspond to a sign of disease, but it is important to consider that this attribute is naturally decreased during larval development [45]. With the application of MB-mediated aPDT, it was possible to observe differences in the attributes of the health index. There was an increase in movement activity and cocoon formation and a reduction in the melanization process, indicating an improvement in the health index that resulted in an increase in survival rate.

In summary, aPDT mediated by the FTC or MB was widely explored in this study, using in vitro and in vivo assays. In the in vitro aPDT, FTC was able to reduce the bacterial growth of *A. baumannii* in planktonic cells, although its effects were less pronounced than MB. Unfortunately, FTC-mediated aPDT was not effective in inhibiting *A. baumannii* in biofilms and in burn injuries as MB had. Seeking to understand the possible action mechanisms involved in these results, we quantified the capacity of FTC and MB to penetrate the *A. baumannii* cells. Indeed, the cellular absorption of MB was significantly higher than FTC, suggesting that FTC needs to be conjugated to cationic polymers or encapsulated in drug delivery systems. These strategies can increase the capacity of FTC to penetrate the cell wall of Gram-negative bacteria that possess a double membrane structure containing numerous strongly negatively charged molecules such as LPS [46].

In conclusion, MB as a photosensitizer in the aPDT showed greater antimicrobial efficacy than FTC against *A. baumannii* strains in both planktonic and biofilm states. MB-mediated aPDT also presented antibacterial activity in burn infections infected by *A. baumannii*. However, aPDT mediated by FTC was not effective in the treatment of these injuries in *G. mellonella*. 

## 4. Materials and Methods

### 4.1. Strains of A. baumannii

Two multidrug-resistant clinical strains of *A. baumannii* H718 (A1) and HC656 (A4) obtained from the Bioclin Laboratory of São José dos Campos-SP were used. The identification and antibiogram of the strains were determined by the semi-automated MicroSCAN 4 system (Beckman Coulter, Indianapolis, IN, USA), which uses broth microdilution, biochemical reactions, and optical density reading. As a reference, a standard strain of *A. baumannii* (ATCC 19606) provided by the National Institute for Quality Control in Health (INCQS) of the Oswaldo Cruz Foundation (FIOCRUZ) was used. These strains were stored in frozen stocks in BHI broth containing 20% glycerol at −80 °C. To activate the microorganisms, *A. baumannii* strains were grown on MacConkey agar culture medium and incubated for 24 h at 37 °C in a bacteriological incubator. Then, colonies characteristic of *A. baumannii* were transferred to the BHI broth and incubated in a bacteriological incubator at 37 °C for 24 h.

### 4.2. Preparation of Standardized A. baumannii Suspensions

To perform each experimental test, a standardized suspension of *A. baumannii* at 10^8^ cels/mL was prepared in a spectrophotometer (B582, Micronal, São Paulo, Brazil) with a wavelength of 600 nm (OD 0.15). In order to confirm the number of bacterial cells used, aliquots of serial dilutions of the inoculum were seeded on plates containing BHI agar for 24 h at 37 °C for CFU/mL counting.

### 4.3. Photosensitizers and Light Source

The photosensitizers used in the study were fototicine (FTC) and methylene blue (MB). FTC (Nuevas Tecnologías Científicas, Lianera Asturias, Spain) was donated by the company Nuevas Tecnologías Científicas, at a concentration of 6.8 mg/mL. The chemical structure and absorption spectrum of the FTC were described in the study by Terra-Garcia et al. [22]. MB (Sigma-Aldrich, São Paulo, Brazil) was dissolved in sterilized distilled water. Both photosensitizers were sterilized by membrane filtration with pores of 0.22 µm in diameter (MFS, Dublin, Ireland) and stored in dark conditions.

For the study in planktonic cultures, concentrations of 0.1 mg/mL of MB [33] and 0.4 mg/mL of FTC were used. The same concentrations were used in the tests of photosensitizer absorption, as the microorganisms used in these tests were in planktonic growth. As biofilms are more resistant than planktonic cells, in the biofilm assays, the concentrations of photosensitizers were increased, using a concentration of 0.2 mg/mL for MB and 1.2 mg/mL for FTC. For the in vivo model, the concentrations used in the biofilm were maintained.

The light source used was a device composed of 48 LEDs (IrradLed 48, Biopdi, São Carlos, SP, Brazil) with a wavelength of 660 nm (visible red), power density of 42.8 mW/cm^2^, energy density of 30 J/cm^2^, and exposure time of 700 s. The study groups carried out were: fotoenticine and light (FTC+L+), methylene blue and light (MB+L+), fotoenticine without irradiation (FTC+L-), methylene blue without irradiation (MB+L-), light in the absence of photosensitizer (P-L+), absence of photosensitizer and light (P-L-).

### 4.4. aPDT Application on Planktonic Cultures of A. baumannii

The aPDT assay on planktonic cultures of *A. baumannii* was performed according to the methodology previously described, with some modifications [47]. An aliquot of 100 µL of standardized bacterial suspension and 100 µL of the photosensitizer (MB or FTC) or PBS were added to each well of the 96-well microplates. The microplates were kept for 15 min in agitation (pre-irradiation time) in dark conditions. Subsequently, the LED irradiation was performed according to the parameters already determined. After irradiation, serial dilutions were seeded on BHI agar and incubated for 24 h at 37 °C to count the CFU/mL numbers.

### 4.5. aPDT Application on A. baumannii Biofilms

Biofilms were formed for 48 h on the bottom of a 24-well microplate using *A. baumannii* ATCC 19606. Then, the supernatant was aspirated, and the wells were washed twice with PBS. According to the group, 200 µL of the photosensitizer (FTC or MB) or PBS was added to each well. After the pre-irradiation time of 30 min (in an orbital shaker), the biofilms of the groups with the presence of light were subjected to irradiation with the LED device. The whole experiment was carried out in the dark. The biofilm adhered to the bottom of the plate was removed using an ultrasonic homogenizer (Sonopuls HD 2200, Bandelin Electronic, Berlin, Germany) with a power of 7 W for 30 s to disaggregate the biofilm. Serial dilutions were seeded on BHI agar and incubated for 24 h at 37 °C to count the CFU/mL numbers.

### 4.6. Analysis of the In Vivo Effects of aPDT in a Burn Model of G. mellonella Infected with A. baumannii 

#### 4.6.1. *G. mellonella* Larvae

*G. mellonella* larvae in their final larval stage were used as a host model. The larvae were obtained from the Invertebrates Laboratory of the Science and Technology Institute-ICT, Unesp São José dos Campos. Each experimental group consisted of 10 larvae with a body weight between 250 and 300 mg. During the experiment, the larvae were not fed. For each assay, a control group composed of larvae with no intervention was included in order to control the quality of rearing larvae. 

#### 4.6.2. Induction of Burn Lesion and Infection of *A. baumannii* in *G. mellonella*

The induction of burn injury was performed followed the methodology of Maslova et al. [41], with some modifications. The larvae were placed in sterile 24-well plates and kept in a refrigerator at 4 °C until use. The antisepsis of the larval cuticle was performed with 70% ethanol. Subsequently, a steel instrument was heated to redness and applied to the dorsal portion of the larvae for a period of 4 s. For infection, immediately after the burn, 10 µL of a standardized suspension of *A. baumannii* (ATCC 19606) was locally applied to the lesion.

#### 4.6.3. aPDT in *G. mellonella*

After 30 min of burn injury and infection with *A. baumannii*, the larvae were subjected to aPDT [29]. For this, the larvae were locally treated with 10 µL of photosensitizer (FTC or MB) in the burn wound. Larvae remained for 30 min in the dark to provide a dispersion of the photosensitizer at the site. Then, LED irradiation was applied according to the previously defined parameters. 

The following groups were included: control group (health larvae not treated), health larvae submitted to light (health larvae treated with light alone), larvae with burn and infection (burn infection not treated), burn and infection submitted to MB-mediated aPDT (burn infection + aPDT with MB), burn and infection submitted to FTC-mediated aPDT (burn infection + aPDT with FTC). Each group consisted of 10 larvae, totaling 50 larvae. Assays were performed in duplicate.

#### 4.6.4. Survival Curve of *G. mellonella* Larvae

After performing the aPDT, the larvae were placed in 24-well plates, incubated at 37 °C in the dark, and analyzed daily over the course of 120 h (5 days). The number of dead larvae was recorded daily to carry out the survival curve. Larvae that did not show any movement to the touch of a metallic tweezer were considered dead. 

#### 4.6.5. *G. mellonella* Larvae Health Index

Larvae were monitored according to a pathological scoring system proposed by Loh et al. [48] for the following attributes: movement activity, extent of silk production (cocoon formation), melanization, and survival. A score was provided for each attribute (Movement activity: 0, no movement; 1, minimal movement with stimulation; 2, movement with stimulation; 3, movement without stimulation. Cocoon formation: 0, without cocoon; 0.5, incomplete; 1, complete cocoon. Melanization: 0, complete; 1, dark points in brown larva; 2, more than three points in beige larva; 3, less than three points in beige larva; 4, no melanization. Survival: 0, dead larva; 2, alive. All the scores together corresponded to a general index of larva health. The average scores obtained for each analyzed attribute were transformed into percentages of 100% and graphically represented. 

### 4.7. Analysis of Photosensitizer Absorption by A. baumannii Cells

This analysis was performed following the methodology proposed by George, Kishen [49], with some modifications. *A. baumannii* (ATCC 19606) were cultivated in BHI broth for 24 h. The growth was centrifuged at 5000 rpm for 10 min and washed with distilled water. The optical density of the cultures was adjusted to 10^8^ cells/mL of *A. baumannii*. After that, 200 μL of this *A. baumannii* suspension was placed in a microtube containing 200 μL of photosensitizer or distilled water, according to the experimental groups. Microtubes were taken to an orbital incubator at 120 rpm for 15 min at 37 °C. After this period, the *A. baumannii* cells were centrifuged at 5000 rpm for 10 min and washed twice with distilled water and lysed with 200 μL of 2% Sodium Dodecyl Sulfate (SDS) for 16 h. The absorbance intensity of the supernatant solution after centrifugation (5000 rpm for 10 min) was measured using a spectrophotometer at the wavelength of 600 nm (B582, Micronal, São Paulo, Brasil). The results were recorded in optical density (OD) values.

### 4.8. Analysis of the Internalization of Photosensitizers by A. baumannii Using Confocal Microscopy

In this assay, 15 mL tubes containing a standardized suspension of *A. baumannii* were centrifuged, and the supernatant was discarded. A total of 100 µL of photosensitizer (FTC or MB) were added, and the tubes were kept for 15 min at room temperature in the dark. Then, washing with PBS was performed, and the sample was resuspended in 200 µL of 4% formol (Ecibra, São Paulo, SP, Brazil) for fixation. After fixation, 100 µL of this solution were deposited per well in 24-well microplates (Kasvi, São José dos Pinhais, PR, Brazil) containing circular coverslips (Knittel Glass, Germany) previously treated with poly-L-lysine (Sigma-Aldrich, São Paulo, SP, Brazil). The microplates were stored in a refrigerator at 4 °C for 18 h. After this period, 15 µL of prolongTM diamond antifade mount with DAPI (InvitrogenTM, São Paulo, SP, Brazil) were used to mount the coverslips under glass slides. The slides were kept in the dark for 18 h for drying and reading. A confocal microscope LSM 700 (Zeiss, Oberkochen, Germany) was used for analysis with excitation at a length of 405 nm for DAPI, 555 nm for AM, and 488 nm for FTC.

### 4.9. Statistical Analysis

Planktonic culture tests, biofilms, and health index of *G. mellonella* larvae were analyzed by the analysis of variance (ANOVA) followed by the Tukey’s test. For the survival curve assays in *G. mellonella*, the Kaplan–Meier method was used, and the level of significance was calculated using the Log-rank test (Mantel–Cox). In all experimental tests, the GraphPad Prism 9.1 program was used, considering a significance level of 5%.

## 5. Conclusions

The new photosensitizer derivative from chlorin e-6, fotoenticine, had antimicrobial activity in the aPDT against *A. baumannii* planktonic cells; however, its effectiveness was lower than aPDT mediated by methylene blue. The antimicrobial effects of fotoenticine and methylene blue occurred when aPDT was applied on reference and multidrug-resistant strains. For both photosensitizers, aPDT showed reduced effectiveness against biofilms compared to planktonic growth. Only methylene blue-mediated aPDT led to significant bacterial reductions of *A. baumannii* biofilms. These results were expanded to in vivo studies, in which a burn infection model was established in *G. mellonella*. This model provided a local application of aPDT that resulted in an improvement of the superficial *A. baumannii* infection when the methylene blue was used as a photosensitizer, confirming the in vitro results. The greatest activity of aPDT mediated by methylene blue was associated with the higher capacity of *A. baumannii* cells in absorbing the methylene blue in relation to fotoenticine. Overall, these results incite future studies focused on new strategies that can increase the capacity of photosensitizers derivative from chlorin to penetrate the cell wall of Gram-negative bacteria, such as the development of functionalized photosensitizers and delivery systems. Certainly, the burn infection model of *G. mellonella* can be a useful tool in the discovery of new strategies to overcome the limitations of aPDT on Gram-negative bacteria, such as *A. baumannii*. 

## Data Availability

Not applicable.
